# The pulmonary and autonomic effects of high-intensity and low-intensity exercise in diesel exhaust

**DOI:** 10.1186/s12940-018-0434-6

**Published:** 2018-12-13

**Authors:** Luisa V. Giles, Christopher Carlsten, Michael S. Koehle

**Affiliations:** 1 Sport Science Department, Douglas College, 700 Royal Ave, New Westminster, BC V3M 5Z5 Canada; 2 School of Kinesiology, University of British Columbia, Vancouver, British Columbia Canada; 3 Department of Medicine, University of British Columbia, Vancouver, British Columbia Canada; 4 Institute for Heart and Lung Health, Vancouver, British Columbia Canada; 5 School of Population and Public Health, University of British Columbia, Vancouver, British Columbia Canada; 6 Division of Sports Medicine, University of British Columbia, Vancouver, British Columbia Canada; 7 Department of Biomedical Physiology and Kinesiology, Simon Fraser University, Burnaby, British Columbia Canada

**Keywords:** Air pollution, Exercise, Pulmonary function, FeNO, Norepinephrine, Exercise intensity

## Abstract

**Background:**

Exposure to air pollution impairs aspects of pulmonary and autonomic function and causes pulmonary inflammation. However, how exercising in air pollution affects these indices is poorly understood. Therefore, the purpose of this study was to determine the effects of low-intensity and high-intensity cycling with diesel exhaust (DE) exposure on pulmonary function, heart rate variability (HRV), fraction of exhaled nitric oxide (FeNO), norepinephrine and symptoms.

**Methods:**

Eighteen males performed 30-min trials of low-intensity or high-intensity cycling (30 and 60% of power at VO_2peak_) or a resting control condition. For each subject, each trial was performed once breathing filtered air (FA) and once breathing DE (300μg/m^3^ of PM_2.5_, six trials in total). Pulmonary function, FeNO, HRV, norepinephrine and symptoms were measured prior to, immediately post, 1 h and 2 h post-exposure. Data were analyzed using repeated-measures ANOVA.

**Results:**

Throat and chest symptoms were significantly greater immediately following DE exposure than following FA (*p* < 0.05). FeNO significantly increased 1 h following high-intensity exercise in DE (21.9 (2.4) vs. 19.3 (2.2) ppb) and FA (22.7 (1.7) vs. 19.9 (1.4)); however, there were no differences between the exposure conditions. All HRV indices significantly decreased following high-intensity exercise (*p* < 0.05) in DE and FA. The exception to this pattern was LF (nu) and LF/HF ratio, which significantly increased following high-intensity exercise (*p* < 0.05). Plasma norepinephrine (NE) significantly increased following high-intensity exercise in DE and FA, and this increase was greater than following rest and low-intensity exercise (*p* < 0.05). DE exposure did not modify any effects of exercise intensity on HRV or norepinephrine.

**Conclusions:**

Healthy individuals may not experience greater acute pulmonary and autonomic effects from exercising in DE compared to FA; therefore, it is unclear if such individuals will benefit from reducing vigorous activity on days with high concentrations on particulate matter.

**Electronic supplementary material:**

The online version of this article (10.1186/s12940-018-0434-6) contains supplementary material, which is available to authorized users.

## Background

In healthy individuals, exercise causes bronchodilation [1] and decreases the fraction of exhaled nitric oxide (FeNO), which is a surrogate measure of pulmonary inflammation [2]. In contrast, air pollution exposure that deposits particulate matter (PM) in the respiratory tree results in pulmonary oxidative stress and an increase in bronchial responsiveness, airway resistance, and airway inflammatory cells [3, 4]. In susceptible populations [5], and some healthy populations [6] such physiological changes due to air pollution exposure can impair pulmonary function. Despite the opposing effects of exercise alone and air pollution exposure alone on pulmonary function and pulmonary inflammation, it is unclear how exercise modifies the pulmonary effects of air pollution.

Exposure to PM can also perturb the autonomic nervous system, resulting in an increase in sympathetic, and a decrease in parasympathetic nervous system activity [7], with a concomitant increase in norepinephrine levels in the paraventricular nucleus of the hypothalamus [8]. At the onset of physical activity, parasympathetic activity decreases [9–15], which likely represents the vagal withdrawal that occurs with physical activity or exercise [16]. Following exercise, perturbations in autonomic control return close to resting levels within 1 h [17]. Diesel exhaust (DE) contains PM and exposure to DE prior to exercise increases exercise heart rate and attenuates exercise-induced bronchodilation [1]. Therefore, it is possible that exposure to DE containing PM during exercise could affect autonomic and pulmonary function. However, there are no studies examining how the effects of DE on the autonomic nervous system are modified by exercise. Additionally, the studies directly examining the effects of continuous exercise with exposure to PM on pulmonary function, have led to inconsistent findings [18–20]. As exercise protocol, duration, and air pollution exposure characteristics vary, drawing any robust conclusions on how exposure to air pollution during exercise affects pulmonary function or inflammation is difficult.

When exercise intensity increases, minute ventilation increases and the proportion of PM that deposits in the respiratory tree increases [21–23], leading to a theoretical increase in the dose of diesel exhaust particulate matter (DPM). Therefore, one might expect that the magnitude of physiological and health effects of air pollution would be greater than during rest or lower intensity exercise. Therefore, the purpose of this study was to determine the effects of low- and high-intensity cycling on pulmonary function, pulmonary inflammation, autonomic function and symptoms of the throat/chest, nose and eyes. We hypothesized that exposure to DE would impair pulmonary function, increase the FeNO, plasma norepinephrine, and subjective symptoms and alter autonomic nervous system function (as measured by heart rate variability (HRV)). We also hypothesized that any physiological effects of DE would be magnified as exercise intensity increases.

## Methods

Eighteen recreationally active males volunteered for the study. Participants were considered recreationally active and included in the study if they met Canada’s physical activity guidelines of 150 min of moderate-to-vigorous activity per week. Only males were studied because FeNO is affected by sex hormones [24] that vary across the menstrual cycle; given that the study required participants to attend on seven occasions, testing in females would have needed to have occurred over a 7-month period, during which time activity levels and physiological parameters could vary significantly leading to an increase in variability of the data. Each participant was a non-smoker and had no history of respiratory or cardiovascular disease. The Clinical Research Ethics Board at the University of British Columbia approved this study. Participants attended an orientation session followed by a reflection period before signing the written informed consent. Prior to all visits, participants were asked to refrain from exhaustive exercise and alcohol for 24 h, caffeine for 6 h, and food or non-water beverages for 2 h. Each participant performed all trials at the same time of day. Participants were also asked to maintain the same pre-test routine including the same mode of travel to the laboratory and pre-test meal, and were asked to refrain from vitamin supplementation for the duration of the study. The sample size was calculated based on a minimal detectable difference in FeNO of 2 ppb [25], using an effect size of 0.37 (Cohen’s *d*), a power of 0.8, and an alpha of 0.05.

### Experimental design

Data collection for this study occurred as part of a larger study and overall methods are explained in detail in other publications [26, 27]. Briefly, each participant attended the laboratory on seven occasions and the initial visit served for familiarization and maximal exercise testing. On the remaining testing days, participants performed 30-min trials of low-intensity cycling, high-intensity cycling, or rest. Each intensity, including rest, was performed once in filtered air (FA) and once in DE with a target concentration of 300 μg/m^3^ of PM_2.5_, for a total of six trials, each of which was separated by a 7-day period. Exercise intensity and the exposure (FA and DE) were randomized with both the participant and the researcher blinded to the exposure condition and data suggests that individuals cannot determine whether they are exposed to DE or FA [28].

#### Introductory session (day 1)

For the maximal exercise test, the cycling work rate started at 100 W and increased by 0.5 W/s until volitional exhaustion. To exclude those subjects with possible exercise-induced bronchoconstriction, any individual with a post-exercise decrease in forced expiratory volume in 1 s (FEV_1_) by 10% or greater was excluded from the study.

#### Testing visits (days 2–7)

Testing visits 2–7 consisted of 30 min trials of cycling or 30 min of rest. Work rates on cycling days were based on the peak power achieved during the maximal exercise test. Low-intensity cycling was set at 30% of the power at ${\overset{\bullet }{V\mathrm{O}}}_{2\mathrm{peak}}$ (96.1 (17.7) W) and high-intensity cycling was set at 60% of power at ${\overset{\bullet }{V\mathrm{O}}}_{2\mathrm{peak}}$ (192.2 (35.3) W), which represented 48 and 77% of ${\overset{\bullet }{V\mathrm{O}}}_{2\mathrm{peak}}$ for low- and high-intensity cycling respectively. Control exposures involved sitting for the same period (30 min), but without performing exercise. Pulmonary function, FeNO, heart rate variability (HRV), plasma norepinephrine and symptoms were measured prior to, immediately post, 1 h, and 2 h post-exposure. During the post exercise/exposure period participants were in a laboratory with normal environmental conditions.

#### Exercise apparatus

Exercise tests were performed using a Velotron cycle ergometer (Racermate Inc., Seattle, WA, USA). During trials, participants breathed through a facemask (7450 Series, Hans Rudolph Inc., Kansas City, MO, USA) attached to a low-resistance, non-rebreathing valve (NRB 2700, Hans Rudolph Inc., Kansas City, MO, USA). Participants remained outside the environmental booth but were connected to the booth via 3.2 cm diameter hoses at both the inspired and expired sides of the non-rebreathing valve.

### Outcome measures

#### Heart rate variability

Following 20 min of supine rest, heart rate was recorded for five min in 15 participants in a quiet, dark room (Polar S810, Polar Electro, Finland). Heart rate variability was analyzed offline using custom software (Kubios HRV, Kuopio, Finland). Time domain measures included the standard deviation of normal-to-normal (NN) intervals (SDNN), the root mean square of the mean differences in successive N-N intervals (RMSSD), and the HRV triangular index. Frequency domain analysis was performed using autoregressive modelling to determine the spectral powers at low frequency (LF: 0.04–0.15 Hz) and high frequency (HF: 0.15–0.40 Hz) as well as total power. Additionally, LF normalized units (LFnu), HF normalized units (HFnu), and LF/HF ratios were determined. Of the 360 measures of HRV taken, seven were excluded due to a poor signal. To prevent complete exclusion of those subjects with missing measurements and based on the recommendations of a statistician, the missing values were imputed using regression [29].

#### FeNO

The FeNO was measured with a NIOX MINO® Airway Inflammation Monitor (Aerocrine, Solna, Sweden), which detects exhaled NO as an indicator of inflammation. Measurements were performed as per the American Thoracic Society guidelines [30]. Briefly, subjects inhaled to close to total lung capacity and then exhaled at a flow rate of 50 ml/s and a pressure of 10 cm H_2_O into the device for 6 s. The device collected the expired gas from the last 3 s of the exhalation to determine the concentration of exhaled NO.

#### Pulmonary function

Pulmonary function was measured in 17 participants using a portable spirometer (Spirobank G, Medical International Research, Rome, Italy), as per the guidelines of the American Thoracic Society [31]. Standard indices of pulmonary function such as forced vital capacity (FVC), FEV_1_, ratio of FVC to FEV_1_ (FEV_1_/FVC), forced expiratory flow during the mid-portion (25–75%) of an FVC (FEF_25–75_) and peak expiratory flow rate (PEFR) were measured. Participants performed three manoeuvres (and up to a maximum of 6, if necessary) per testing time point. For repeatability to be achieved, the difference between the highest and second highest trial for FEV_1_ and FVC was required to fall within 0.15 L. The peak value for each variable was used for analysis.

#### Plasma norepinephrine

Blood samples were taken from the right antecubital fossa with a 21-gauge needle into vacutainers containing EDTA. All blood samples were immediately centrifuged at 1500 *g* for 20 min to separate plasma from formed elements. Plasma was extracted, frozen, and stored at − 80 °C until assayed. Plasma concentrations of norepinephrine were determined using commercially available enzyme-linked immunosorbent assay (ELISA) kits (Norepinephrine ELISA kit, Abnova, CA, USA) and according to the procedures outlined by the manufacturer. Plasma levels of norepinephrine were measured using a Versa Max microplate reader (Molecular Devices Corporation, CA, USA). The intra assay coefficient of variation for norepinephrine was 7.1%.

Levels of norepinephrine were adjusted for changes in plasma volume from baseline. The estimated post-exercise concentration of markers due to plasma volume changes alone was estimated using the following equation [32]:

$$ {Concentration}_{ESTIMATED}=\frac{Hct_{POST}\times \left(100-{Hct}_{PRE}\right)}{Hct_{PRE}\times \left(100-{Hct}_{POST}\right)}\times {Concentration}_{PRE} $$

where *Hct* represents hematocrit. The adjusted concentration was then calculated using the following equation:

$$ {Concentration}_{ADJUSTED}=\left({Concentration}_{PRE}-{Concentration}_{ESTIMATED}\right)+{Concentration}_{MEASURED} $$

#### Symptoms

At each time point, participants were asked to rate symptoms on a scale of 0–5 with 0 being “no symptoms” and 5 being “severe”. Participants were blinded to scores from previous time points and test days. Symptoms were grouped into eyes, nose, throat/chest and other and included the following questions:

*Eye symptoms:* Are your eyes itchy? Are your eyes watering? Do you feel a painful or stinging sensation in your eyes?

*Nasal symptoms:* Does your nose feel itchy? Do you feel a painful or stinging sensation in your nose? Is your nose running? Is your nose blocked? Are you sneezing?

*Throat/Chest symptoms:* Does your throat feel dry, scratchy, or sore? Are you wheezing or do you have any whistling sounds in your chest? Are you having any chest pain? Are you having chest tightness? Do you have shortness of breath?

*Other symptoms:* Do you have a headache? Do you feel fatigued? Do you feel nauseous?

Within each category scores from individual questions were summed to provide a score for nasal, eyes, throat/chest and other. The symptoms chosen were those typically reported in similar studies which demonstrate that participants are unable to determine if they are exposed to DE or FA [28].

### Exposure setup

All exposures were performed using an environmental exposure booth that is explained in detail elsewhere [33], but modified only in that load was constant at 2.5 kW. For DE exposures, participants were exposed to calibrated, aged, and diluted DE that had a target concentration of 300 μg/m^3^ of PM_2.5_. In-booth PM mass concentration measurements were made using a Tapered Element Oscillating Microbalance (TEOM; Model 1400a, Rupprecht & Pattashnick, Albany, NY, USA) using 10 min averages. A TSI Scanning Mobility Particle Scanner (Model 3936, TSI, Shoreview, MN, USA) classified the particle size distribution between 2.5 nm and 1000 nm. For FA exposures, participants were exposed to compressed, HEPA-filtered air.

### Statistical analysis

Statistical analyses were completed using SPSS software (SPSS Inc., version 20, Chicago, IL) and analyses were chosen through consultation with a PhD statistician. For each parameter, data were analyzed using a 2 (exposure: FA vs. DE) × 3 (intensity: rest, low-intensity, high-intensity) × 4 (time: pre, post, 1 h, 2 h) repeated measures ANOVA. Significance was set at *p* < 0.05. Main or interaction effects were further analyzed using pair-wise comparisons and significance was adjusted to account for multiple comparisons using the Sidak adjustment. The *p*-values represented in this manuscript have been inflated to incorporate the Sidak adjustment, meaning that α remains at 0.05. Specifically, the Sidak adjustment uses the following equation to adjust for multiple comparisons: 1- (1-unadjusted *p*-value)^1/k^, where k is the number of comparisons in the family. For the post-hoc analysis comparing exercise intensity, *p*-values were adjusted for 3 groups (rest, low-intensity and high-intensity) and when time was compared, *p*-values were adjusted for 4 groups (pre, post, 1 h, 2 h). All means are reported with standard deviations in parentheses.

## Results

Eighteen recreationally active males (age 24.5 (6.2) yr. (mean (sd)); height: 1.78 (0.08) m; body mass: 74.2 (10.5) kg) completed the study. Their mean ${\overset{\bullet }{V\mathrm{O}}}_{2\mathrm{peak}}$ was 55.0 (9.1) mL•kg^− 1^•min^− 1^, participants had a mean maximum power output was 320.4 (58.9) W, and mean maximum heart rate was 182.1 (12.7) bpm.

Baseline outcome variables were not significantly different across the six test days (*p* > 0.05). All participants performed all six trials, although three participants were unable to finish the high-intensity trial in DE due to volitional exhaustion. In individuals who were unable to finish the first high-intensity trial, the second high-intensity exercise trial was designed to mimic the first; therefore, the duration in trial two was reduced to match the first trial. Exposure to PM_2.5_ was 9.3 (6.20) and 302.1 (6.50) μg/m^3^ for FA and DE respectively. Mean particle number concentration (PNC) during FA and DE exposures were 0.14 × 10^4^ and 61.60 × 10^4^ (#/cm^3^). Mean NO_2_ during FA and DE exposures was 0.04 (0.04) and 0.58 (0.15) ppm. Mean NO during FA and DE exposures was 0.02 (0.02) and 7.00 (0.09) ppm. Mean carbon monoxide during FA and DE exposures was 3.00 (0.40) and 13.9 (2.10) ppm.

**Fig. 1:**
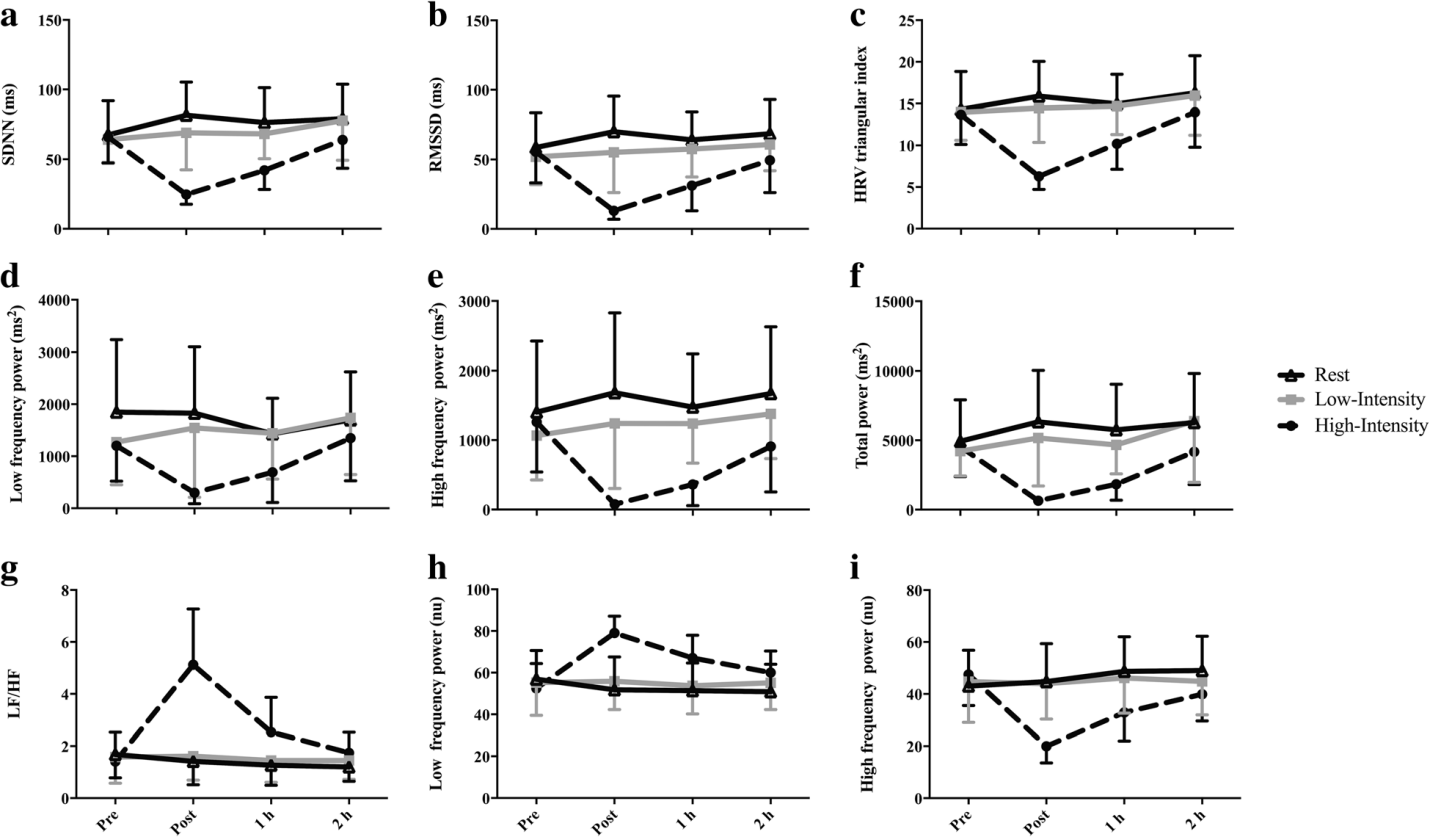
Heart rate variability (HRV) in 15 males prior to and following rest, low-intensity, or high-intensity cycling: (**a**) SDNN: Standard deviation of NN intervals, (**b**) RMSSD: root mean square of successive intervals, (**c**) HRV triangular index, (**d**) Low frequency (LF) power, (**e**) High frequency (HF) power, (**f**) Total power, (**g**) LF/HF, (**h**) LF power normalized units (nu), (**i**) HF power nu. A summary of significant differences can be found in Additional files 1 and 2

There was a significant intensity-by-time interaction for all time and frequency domain indices of HRV (*p* < 0.05). All indices except LF (nu) and LF/HF ratio significantly decreased following high-intensity exercise. Immediately post and 1 h post-exposure, these variables were significantly lower following high-intensity exercise compared to following rest. Conversely, compared to baseline and to rest, LF (nu) and LF/HF ratio significantly increased following high-intensity exercise (Fig. 1). For detailed comparisons of significant differences see Additional files 1 and 2. There were no effects of exposure condition on any indices of HRV.

**Fig. 2:**
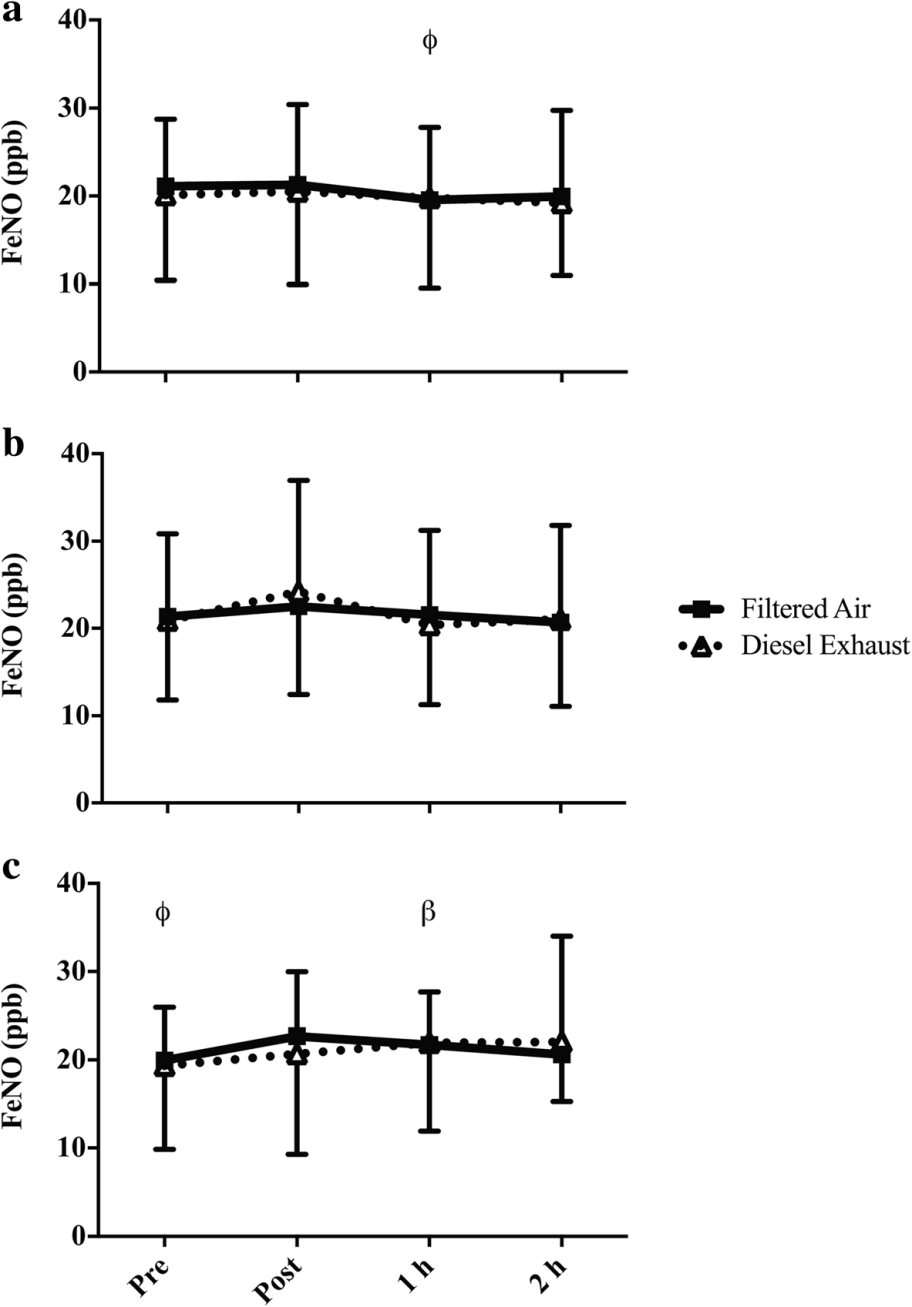
FeNO prior to and following **a** rest, **b** low-intensity, or **c** high-intensity cycling in FA or DE. Significance is set at *p* < 0.05. β = significantly greater than pre-exercise in the corresponding exposure, occurs only in DE (diesel exhaust) only; ϕ = significantly less than post in the corresponding exposure (FA (filtered air) only)

There was a significant three-way interaction for FeNO (exposure-by-intensity-by-time; *p* = 0.045, Fig. 2). Immediately following rest in FA, FeNO was significantly greater than at 1 h post exposure (Fig. 2a. *p* = 0.025; 21.3 (2.2) ppb vs. 19.6 (2.0) ppb). One hour following high-intensity exercise in DE, FeNO was significantly greater than pre-exercise (Fig. 2c. *p* = 0.024; 21.9 (2.4) ppb, vs. 19.3 (2.2) ppb). Prior to high intensity exercise in FA, FeNO was significantly less than immediately post exposure (Fig. 2c. *p* = 0.048; 19.9 (1.4) ppb, vs. 22.7 (1.7) ppb); however, there were no differences between FA and DE for any comparisons.

**Fig. 3:**
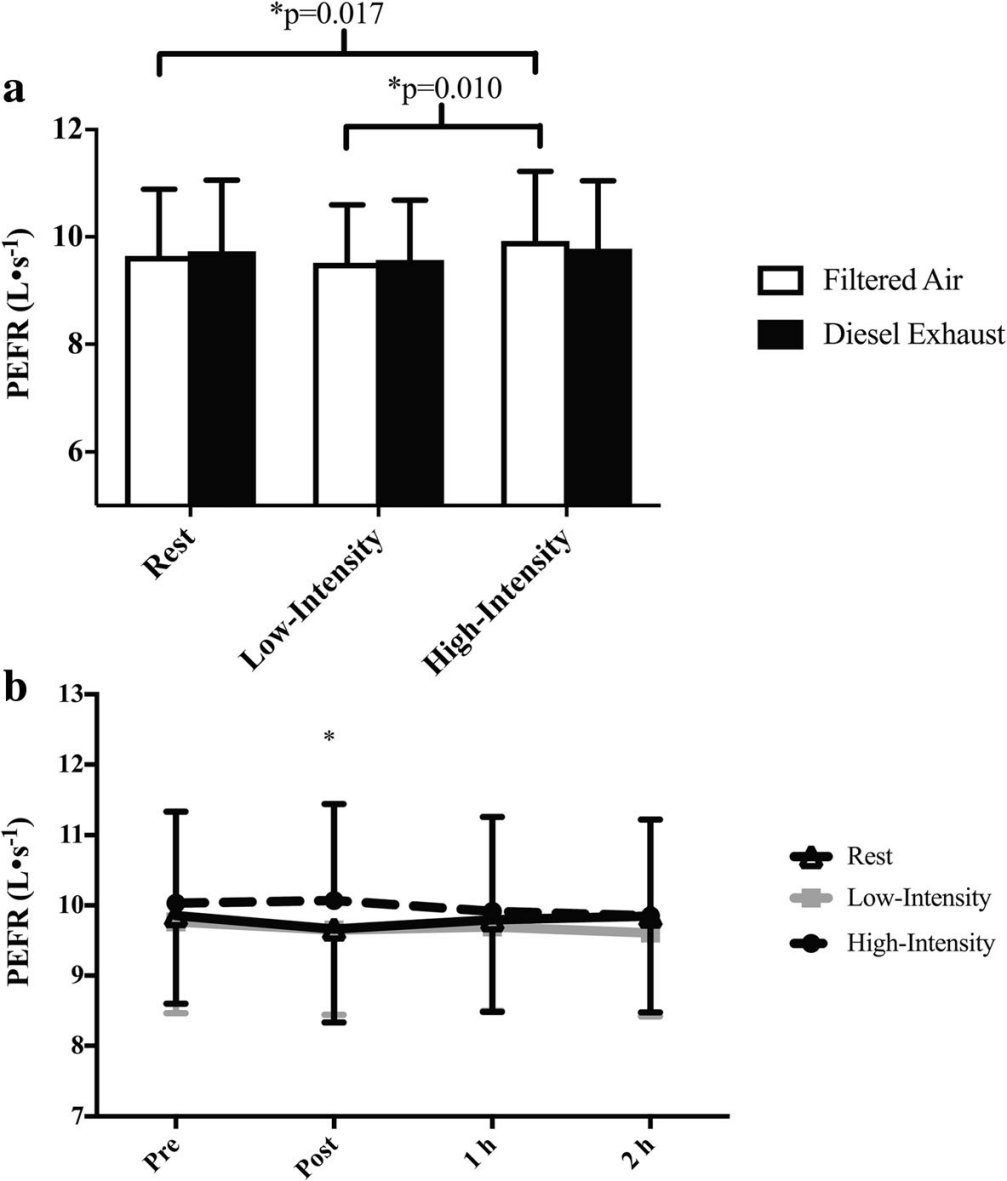
**a** Exposure-by-intensity interaction and **b** intensity-by-time interaction for PEFR. Significance is set at *p* < 0.05. For the exposure-by-intensity interaction data is collapsed across all time points and for the intensity-by-time interaction data is collapsed across both exposure conditions. * Low-intensity (*p* = 0.011) and rest (*p* = 0.017) are significantly less than high-intensity exercise at the post-exercise time point

Mean baseline FEV_1_ was 4.31 (0.76) L, FVC was 5.48 (1.13) L, FEV_1_/FVC was 0.79 (0.07), FEF_25–75_ 4.00 (0.91) L/s and PEFR was 9.79 (1.31) L/s. There was a significant interaction effect for PEFR (exposure-by-intensity: *p* = 0.036, Fig. 3a; intensity-by-time: *p* = 0.04, Fig. 3b). An exposure-by-intensity interaction suggested that during high-intensity exercise in FA, PEFR was significantly greater than low-intensity exercise (Fig. 3a. *p* = 0.010; 9.93 (1.30) L/s vs. 9.52 (1.08) L/s) and rest (*p* = 0.017; 9.93 (1.30) L/s vs. 9.65 (1.24) L/s). However, this relationship did not occur with DE. An intensity-by-time interaction showed that immediately following high-intensity exercise PEFR was significantly greater than following low-intensity exercise (Fig. 3b. *p* = 0.011; 9.95 (1.30) L/s vs. 9.52 (1.11) L/s) and rest (*p* = 0.017; 9.95 (1.30) L/s vs. 9.57 (1.31) L/s). There were no other main or interaction effects for FEV^1^, FVC or FEF^25–75^.

**Fig. 4:**
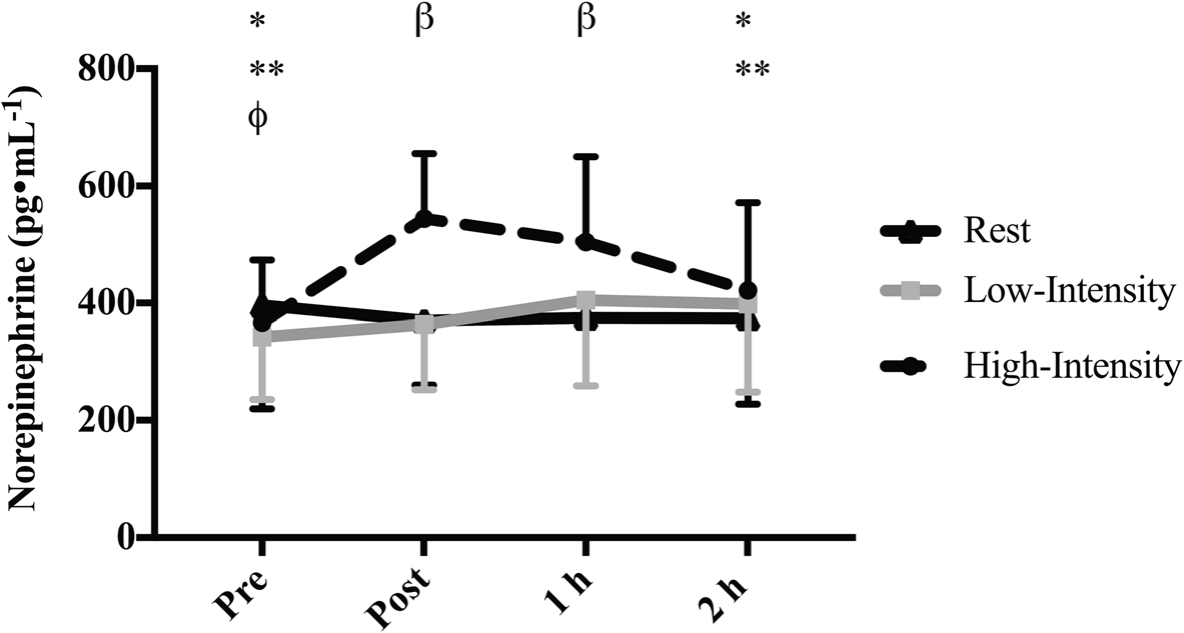
Plasma norepinephrine in 18 males prior to and following rest, low-intensity cycling and high-intensity cycling. Significance is set at *p* < 0.05. For the intensity-by-time interaction data is collapsed across both exposure conditions. * Significantly less than post in the corresponding exercise intensity (high-intensity only). ** Significantly less than 1 h in the corresponding exercise intensity (high-intensity only). *β* Significantly greater in high-intensity compared to low-intensity and rest. Φ Significantly less than 1 h in the corresponding exercise intensity (low-intensity only)

There was a significant interaction effect (intensity-by-time: *p* < 0.001, Fig. 4) for plasma norepinephrine but DE exposure did not modify this response. One hour post low-intensity exercise norepinephrine levels were significantly greater than prior to exercise (*p* = 0.015; 404.75 (34.43) pg•mL^− 1^ vs. 342.59 (25.24) pg•mL^− 1^). Prior to high intensity exercise norepinephrine was significantly less than immediately post (*p* = 0.003; 366.69 (29.81) pg•mL^− 1^ vs. 544.23 (39.25) pg•mL^− 1^) and 1 h post exercise (*p* = 0.002; 366.69 (29.81) pg•mL^− 1^ vs. 504.16 (31.49) pg•mL^− 1^). The elevation seen immediately post and 1 h post high-intensity exercise meant that plasma norepinephrine levels at these time points were significantly greater than 2 h post exercise (*p* = 0.007; post vs. 2 h: 544.23 (39.25) pg•mL^− 1^ vs. 421.71 (25.13) pg•mL^− 1^; *p* = 0.007; 1 h vs. 2 h: 504.16 (31.49) pg•mL^− 1^ vs. 421.71 (25.13) pg•mL^− 1^). Norepinephrine was significantly greater immediately post high-intensity exercise compared to immediately post rest (p = 0.002; 544.23 (39.25) pg•mL^− 1^ vs. 370.10 (25.83) pg•mL^− 1^) and low-intensity exercise (*p* < 0.001; 544.23 (39.25) pg•mL^− 1^ vs. 363.81 (26.31) pg•mL^− 1^). Similarly, norepinephrine was significantly greater 1 h following high-intensity exercise compared to 1 h post rest (*p* = 0.004; 504.16 (31.49) pg•mL^− 1^ vs. 375.17 (27.37) pg•mL^− 1^) and low-intensity exercise (*p* = 0.025; 504.16 (31.49) pg•mL^− 1^ vs. 404.76 (34.43) pg•mL^− 1^).

**Fig. 5:**
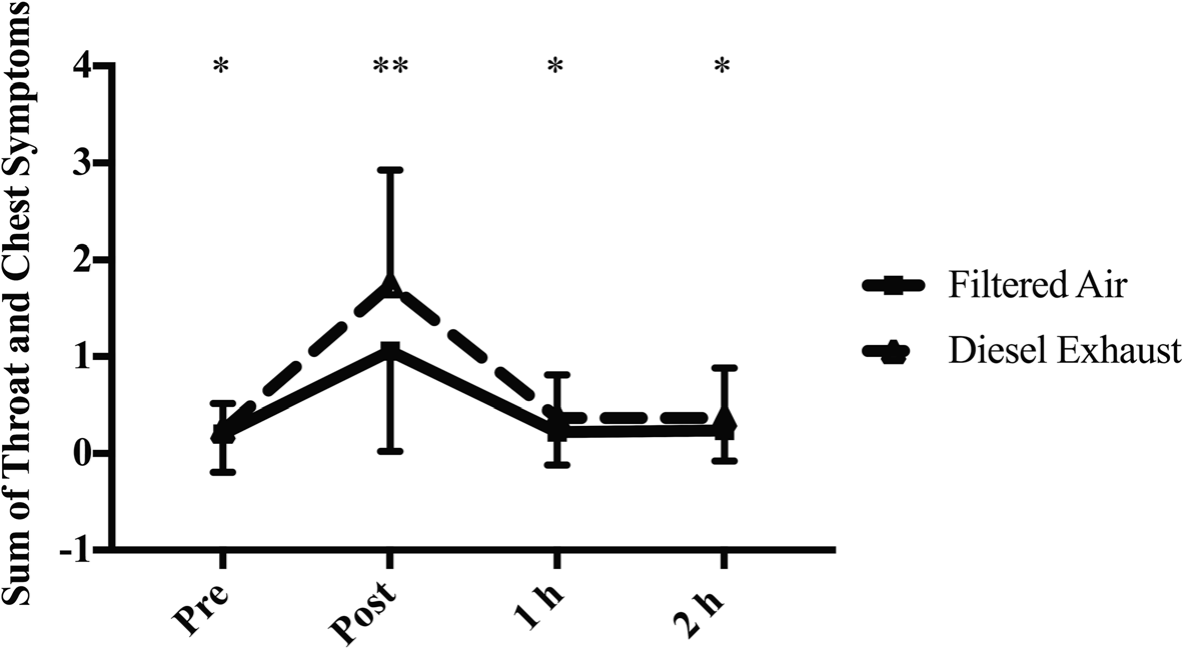
Sum of Throat and Chest symptoms prior to and following FA and DE. Significance set at 0 < 0.05. FA: Filtered air, DE: Diesel exhaust. *Significantly less than post in the corresponding exposure (occurs in both DE and FA). ** DE significantly greater than FA at the same time point

There was a significant interaction effect (condition-by-time: *p* = 0.021, Fig. 5) for throat/chest symptoms. Immediately following exposure to FA, symptoms for the throat/chest were significantly greater than symptoms prior to exposure (*p* = 0.005; 1.06 (0.24) vs. 0.24 (0.07)), 1 h post exposure (*p* = 0.032; 1.06 (0.24) vs. 0.22, (0.08)) and 2 h post exposure (*p* = 0.020; 1.06 (0.24) vs. 0.24 (0.08)). Similarly, immediately following exposure to DE, symptoms for the throat/chest were significantly greater than symptoms prior to exposure (*p* < 0.001; 1.74 (0.28) vs. 0.24 (0.07)), 1 h post exposure (*p* < 0.001; 1.74 (0.28) vs. 0.37 (0.10)) and 2 h post exposure (*p* = 0.001; 1.74 (0.28) vs. 0.37 (0.12)). However, the increase in symptoms immediately following DE was significantly greater following FA (*p* = 0.024; 1.74 (0.28) vs. 1.06 (0.24)).

There was also a significant intensity-by-time interaction for eye symptoms (*p* = 0.042), nasal symptoms (*p* = 0.031), throat/chest symptoms (*p* < 0.001) and other symptoms (*p* < 0.001). Post hoc comparisons for eye symptoms did not reveal significant differences. See Table 1 for Post hoc comparisons for the intensity-by-time interaction for all categories of symptoms. The dataset supporting the conclusions of this can be made available upon request.

**Table 1:** Mean (SD) for subjective symptoms in 18 recreationally active males prior to and following exercise or rest

	Rest	Low-Intensity	High-Intensity
	Mean (SD) [95% CI]		
Nasal Symptoms			
Pre	0.33 (0.45)	0.22 (0.43)*	0.44 (0.48)
Post	0.42 (0.65)**	0.64 (0.76)*	1.08 (1.06)
1 h post	0.25 (0.39)	0.17 (0.30)	0.39 (0.63)*
2 h post	0.19 (0.31)	0.08 (0.26) *	0.17 (0.34)*
Throat/Chest Symptoms			
Pre	0.28 (0.39)*	0.25 (0.43) *	0.14 (0.33) *
Post	0.81 (0.79)**	0.83 (0.66)**	2.56 (2.07)
1 h post	0.39 (0.47)	0.17 (0.34) * Φ	0.33 (0.42) *
2 h post	0.33 (0.42)*	0.31 (0.49) *	0.28 (0.39) *
Eye Symptoms			
Pre	0.36 (0.17)	0.19 (0.11)	0.11 (0.09)
Post	0.17 (0.07)	0.22 (0.08)	0.50 (0.17)
1 h post	0.14 (0.08)	0.11 (0.09)	0.14 (0.07)
2 h post	0.08 (0.06)	0.06 (0.04)	0.11 (0.07)
Other Symptoms			
Pre	0.19 (0.07)	0.22 (0.09)	0.14 (0.07)*
Post	0.47 (0.17)**	0.67 (0.17)* **	2.11 (0.34)
1 h post	0.14 (0.07) **	0.31 (0.11)	0.67 (0.23)*
2 h post	0.11 (0.07) **	0.14 (0.05)	0.31 (0.09)*

*significantly less than post in the corresponding intensity, *p* < 0.05

**significantly less than high-intensity at the corresponding time point, *p* < 0.05

Φ significantly less than rest at the corresponding time point, *p* < 0.05

## Discussion

This is the first study to determine the pulmonary and autonomic nervous system effects of (DE) exposure with exercise of varying intensities. We found that following 30 min of exercise pulmonary function, FeNO, HRV, or plasma norepinephrine were not significantly different between low- and high-intensity exercise in DE. However, exposure to DE did exacerbate throat and chest symptoms to a significantly greater degree than following FA.

Particulate matter exposure causes oxidative stress, and increases bronchial responsiveness, airway resistance, and airway inflammatory cells [3, 4]. Following high-intensity exercise in FA and DE (immediately post in FA and 1 h post in DE) FeNO, which is a surrogate measure of airway inflammation, was significantly greater than compared to baseline. The increase in FeNO was similar between DE and FA, suggesting that DE did not magnify the response. Therefore, these data suggest that in healthy individuals, exercise in DE does not potentiate pulmonary inflammation as measured by FeNO; however, we cannot discount that other markers of pulmonary inflammation, such as eosinophils in sputum, have been affected. The lack of a DE effect contradicts our initial hypothesis that DE exposure would increase the amount of pulmonary inflammation post-exercise. Our findings are similar to others showing that in healthy individuals, acute exercise/ physical activity in a high pollution/high traffic environment is not associated with elevated FeNO [19, 34, 35]. Rundell et al. assessed the FeNO response to 30 min of cycling at 85–95% of maximum heart rate while Jacobs et al. assessed the FeNO response to 20 min of cycling at 74% of maximum heart rate [19, 34]. The intensities used by Rundell et al. and Jacobs et al. are similar to the high- and low-intensity trials in the current study [19, 34] and further corroborate our findings that exercise of varying intensities with exposure to air pollution does not affect FeNO. In this study we did not see acute changes in FeNO with exposure to DE but it is possible that repeated exposure during exercise/ physical activity is necessary to increase FeNO. For example, Bos et al. [36] found that individuals who aerobically trained in a polluted urban environment had significantly elevated FeNO compared to those aerobically training in a less polluted rural environment. The absence of a DE and FA difference could also be due to post-exercise measurements occurring over too short of a time frame or that that FeNO may not be a stable or sensitive enough indicator of pulmonary inflammation in healthy individuals.

Our finding suggesting that high-intensity exercise increases FeNO, contrasts with work done by Verges et al. who examined FeNO following 25 min of incremental exercise (two 10 min bouts at 46 and 60% of peak power, followed by five min at 90% of peak power) [37]. Immediately following exercise, Verges et al. found that FeNO significantly decreased [37]. Similarly, Evjenth et al. tested FeNO following an 8 min incremental test that reached 95% maximum heart rate [2] and found that immediately following and 30 min following exercise, FeNO levels were significantly reduced [2]. Our results may be in opposition to others [2, 37] due to the timeframe of sampling. In the study by Verges et al., FeNO levels were returning towards baseline 15 min following exercise [37]. The measurement of post-exercise FeNO in the current study occurred following a number of other physiological measures, which meant that the post-exercise FeNO measurement could have been measured up to 40 min post-exercise. Therefore, an immediate post-exercise reduction in FeNO could have been missed in the current study. As exercise duration was different between the current study and others [2, 37], it is also possible that a different FeNO response was elicited.

In susceptible populations, PM-induced physiological changes such as oxidative stress, bronchial hyperresponsiveness, and inflammation [3, 4] can result in impaired lung function [38]. Within the current study, we found that in FA, PEFR was significantly greater following high-intensity exercise compared to rest. In DE, these differences did not occur, leading to a trend for lower PEFR in DE compared to FA. As the FA vs. DE differences were not significant, and there were no effects of exposure on other parameters of pulmonary function, one cannot conclude from this study that DE exposure during exercise affects pulmonary function or that exercise intensity magnifies this response. Our findings are similar to those of others who found no associations between exposure to air pollution/PM and pulmonary function following exercise or physical activity in urban areas [18, 20, 35], and found that physical activity reduced PM associated decrements in aspects of lung function such as PEFR [6].

Immediately following DE exposure, throat and chest symptoms were 0.68 points higher on a 5-point scale compared to FA. Since the DE condition had such a high concentration of PM_2.5_, these findings are not surprising. It is interesting that in this healthy cohort, the participants noticed more symptoms, yet the objective measures of lung function and inflammation were not impacted. While, DE exposure contains a mixture of particulate matter and gaseous pollutants, it is possible that we did not observe differences in lung function and inflammation as the mixture within the current study may differ from ambient conditions. Additionally, we may not have observed significant differences based on the marker of pulmonary inflammation chosen. FeNO is a surrogate measure of eosinophilic airway inflammation and thus we cannot discount that other aspects of airway inflammation were impacted. Also, we cannot disregard that exercise duration, the time course of post exercise measures and the fitness level or health status of our participants may have led to non-significant findings. Finally, the sample size for the current study was calculated based on a minimal detectable difference in FeNO; therefore, it is possible that other outcomes measures were not adequately statistically powered.

One of the other potential pathways in which PM exposure causes cardiovascular effects is through an increase in sympathetic nervous system activity and a decrease in parasympathetic nervous system activity demonstrating a disruption in cardiac autonomic control [39]. Contrary to our hypothesis, our research demonstrated that HRV is not impacted by a controlled exposure to DE. This finding is in accordance with other controlled experimental diesel exposure studies [40, 41]. However, some epidemiological research suggests that as PM_2.5_ [42] or PM_10_ [43, 44] concentrations increase, HRV is impaired (as demonstrated by reductions in SDNN and RMSSD), yet there is still is a lack of consensus on the magnitude, direction, and existence of an effect; as is demonstrated by some studies finding a significant but positive association between PM and SDNN [45] and others finding no association between PM and indices of HRV [46]. Despite inconsistencies in the literature, it appears that controlled diesel exposure studies demonstrate a lack of an effect on HRV, while observational and epidemiological studies are generally conflicting. This discrepancy could be related to three factors. DE does not contain metals found in atmospheric air pollution such as nickel, lead, arsenic and cadmium. These metals are thought to play a key role in the autonomic nervous system response to air pollution [47]. A laboratory environment allows researchers control of confounding factors and in this context does not contain extraneous stressors, such as noise and traffic, which may affect HRV. Finally, it is possible that there could be a more long-term association between air pollution and HRV that is not captured in the laboratory studies.

As highlighted, some PM exposure can cause an increase in sympathetic nervous system activity and a decrease in parasympathetic nervous system activity [39]. As both the adrenal medulla and sympathetic nerve endings produce norepinephrine, it is no surprise that production of norepinephrine will increase in response to sympathetic stimulation and potentially PM exposure. Norepinephrine is produced by sympathetic nerve endings and plays an important role in the pulmonary inflammatory response to PM exposure [48]. Therefore we reasoned that any changes in HRV and FeNO would be accompanied by changes in norepinephrine. We found that plasma norepinephrine levels were significantly increased following high intensity exercise, but there was no difference in response between DE and FA. These findings follow a similar pattern to the changes in FeNO and HRV and are likely related to persistent sympathetic activation following exercise/physical activity. The lack of an effect of DE on norepinephrine is similar to other human studies [49], but contrasts with human studies animal studies that found exposure to concentrated ambient particles increases norepinephrine in urine [50] and the paraventricular nucleus of the hypothalamus [8]. As with the HRV findings, the absence of effect of DE on norepinephrine could be related to the constituents of the pollution exposure, the time course of the study, or the lack of extraneous stressors such as noise and traffic.

## Conclusions

The current study assessed the acute pulmonary, autonomic nervous system and symptomatic effects to 30 min of rest, low-, and high-intensity cycling with DE exposure in healthy recreationally active males. We initially hypothesized that DE would impair pulmonary function, cause pulmonary inflammation, increase symptoms related to DE exposure and result in autonomic responses that would be magnified by exercise intensity; however, our results do not support these hypotheses. Based on the results of this study, healthy individuals may experience an increase in throat and chest symptoms with DE exposure but exercising in DE does not magnify these. Additionally, healthy individuals may not experience acute pulmonary and autonomic effects from exercising in DE. However, to substantiate this claim, more research is needed to determine the effects of different compositions of air pollution over longer time frames during exercise. Furthermore, these findings only apply to a healthy, active population; further work in clinical populations is necessary in order to understand the combined effects of air pollution and exercise in these groups.

## Additional files

Additional file 1: Intensity-by-time interaction summary table for heart rate variability, summarizing significant differences (*p* < 0.05) at each intensity between time points. (DOCX 66 kb)

Additional file 2: Intensity-by-time interaction summary table for heart rate variability, summarizing significant differences (*p* < 0.05) at each time point between exercise intensities. (DOCX 72 kb)

## Abbreviations

${\overset{\bullet }{V\mathrm{O}}}_{2\mathrm{peak}}$: Peak oxygen consumption
DE: Diesel exhaust
DPM: Diesel exhaust particulate matter
ELISA: Enzyme-linked immunosorbent assay
FA: Filtered air
FEF_25–75_: Forced expiratory flow in the middle 50% of expiration
FeNO: Fraction of exhaled nitric oxide
FEV_1_: Forced expiratory volume in 1 s
FVC: Forced vital capacity
H: Hour(s)
HF (nu): High frequency power in normalized units
HFP: High frequency power
HRV: Heart rate variability
LF (nu): Low frequency power in normalized units
LF/HF: Low frequency/high frequency ratio
LFP: Low frequency power
Min: Minutes
NE: Norepinephrine
NN: Normal-to-normal interval
NO: Nitric oxide
NO_2_: Nitrogen dioxide
PEFR: Peak expiratory flow rate
PM: Particulate matter
PM_10_: Particulate matter with a mass median aerodynamic diameter less than 10 μm
PM_2.5_: Particulate matter with a mass median aerodynamic diameter less than 2.5 μm
PNC: Particle number concentration
RMSSD: Root mean square of successive intervals
SDNN: Standard deviation of normal-to-normal intervals
UFP: Ultrafine particulate matter
