# Supplementary material for: The pulmonary and autonomic effects of high-intensity and low-intensity exercise in diesel exhaust
Source: Environ Health. 2018 Dec 13;17:87. doi: 10.1186/s12940-018-0434-6 (PMC6292001; doi:10.1186/s12940-018-0434-6)
Supplement: Supplementary file 1 — Intensity-by-time interaction summary table for heart rate variability, summarizing significant differences (p < 0.05) at each intensity between time points. (DOCX 66 kb) [file 12940_2018_434_MOESM1_ESM.docx]

Additional File 1

Intensity-by-time interaction summary table for heart rate variability, summarizing significant differences (p<0.05) at each intensity between time points

| Intensity | Time comparison | HRV time domain | HRV frequency domain |
| --- | --- | --- | --- |
| Rest | Pre vs. Post | SDNN |  |
|  | 1 h vs. 2 h | TRI |  |
| Low-Intensity | Pre vs. 2 h | RMSSD |  |
| High-Intensity | Pre vs. Post | SDNN, RMSSD, TRI | LFP, HFP, Total Power, LF/HF, LF (nu), HF (nu) |
|  | Pre vs. 1 h | SDNN, RMSSD, TRI | HFP, Total Power, LF (nu), HF (nu) |
|  | Post vs. 1 h | SDNN, RMSSD, TRI | LFP, HFP, Total Power, LF/HF, LF (nu), HF (nu) |
|  | Post vs. 2 h | SDNN, RMSSD, TRI | LFP, HFP, Total Power, LF/HF, LF (nu), HF (nu) |
|  | 1 h vs. 2 h | SDNN, RMSSD, TRI | LFP, HFP, Total Power, LF/HF, LF (nu), HF (nu) |

All parameters shown have a p<0.05 after adjustment for multiple comparisons using Sidak. Abbreviations: HF: High frequency; HRV: Heart rate variability; LF: Low frequency; nu: normalized units; RMSSD: root mean square of successive intervals; SDNN: Standard deviation of normal-to-normal intervals
