# Supplementary material for: The pulmonary and autonomic effects of high-intensity and low-intensity exercise in diesel exhaust
Source: Environ Health. 2018 Dec 13;17:87. doi: 10.1186/s12940-018-0434-6 (PMC6292001; doi:10.1186/s12940-018-0434-6)
Supplement: Supplementary file 2 — Intensity-by-time interaction summary table for heart rate variability, summarizing significant differences (p < 0.05) at each time point between exercise intensities. (DOCX 72 kb) [file 12940_2018_434_MOESM2_ESM.docx]

Additional File 2

Intensity-by-time interaction summary table for heart rate variability, summarizing significant differences (p<0.05) at each time point between exercise intensities

| Time | Intensity comparison | HRV time domain | HRV frequency domain |
| --- | --- | --- | --- |
| Post | Rest vs. Low-Intensity | Mean RR, PNN50 |  |
|  | Rest vs. High-Intensity | SDNN, RMSSD, TRI | LFP, HFP, Total Power, LF/HF, LF (nu), HF (nu) |
|  | Low- vs. High-Intensity | SDNN, RMSSD, TRI | LFP, HFP, Total Power, LF/HF, LF (nu), HF (nu) |
| 1 h | Rest vs. High-Intensity | SDNN, RMSSD, TRI | LFP, HFP, Total Power, LF/HF, LF (nu), HF (nu) |
|  | Low- vs. High-Intensity | SDNN, RMSSD, TRI | LFP, HFP, Total Power, LF/HF, LF (nu), HF (nu) |
| 2 h | Rest vs. High-Intensity | RMSSD | LF/HF, LF (nu), HF (nu) |

Pre values were not compared between exercise intensities as a 1-way repeated measures ANOVA was performed to ensure that all pre test values were not significantly different. All parameters shown have a p<0.05 after adjustment for multiple comparisons using Sidak.

Abbreviations: HF: High frequency; HRV: Heart rate variability; LF: Low frequency; nu: normalized units; RMSSD: root mean square of successive intervals; SDNN: Standard deviation of normal-to-normal intervals
